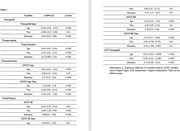# Demographic Correlates of Visuo‐Cognition in a Healthy Ageing Indian Population ‐ A Cross‐Sectional Analysis

**DOI:** 10.1002/alz.091239

**Published:** 2025-01-03

**Authors:** Vindhya Vishwanath, Palash K. Malo, Ajith Partha, Divya N M, Abhishek Mensegere Lingegodwa, Albert Stezin, Amitha C M, Rajitha Narayanasamy, Meenakshi Menon, Meghana R, Goutham Velavarajan, Deva Kumar HS, Prathima Arvind, Monisha S, Shafeeq K Shahul Hameed, Sunitha HS, Deepashri Agrawal, Banashree Mondal, Sadhana Singh, Jonas S. Sundarakumar, Thomas Gregor Issac

**Affiliations:** ^1^ Centre for Brain Research, Indian Institute of Science, Bangalore, Karnataka India; ^2^ Centre for Brain Research, Indian Institute of Science, Bengaluru, Karnataka India

## Abstract

**Background:**

Visuo‐cognitive skills represent a network of different abilities that rely on vision and cognition. While visuo‐cognitive abilities have been considered prominent indicators of dementia, there is a dearth of studies that profile these abilities with demographic correlates in an aging Indian population. Investigating the pattern of visuo‐cognitive abilities is essential to facilitate early indication, better prognosis and treatment of symptoms.

**Method:**

The sample comprises baseline data collected from 1271 urban aging individuals (≥ 45 years) who are a part of the Tata Longitudinal Study on Ageing (TLSA), Bangalore, India. Visuo‐cognition was measured by Visuospatial span (VS), Visual Attention (VA), Modified Taylor Complex Figure (MTCF) Copy, Immediate Recall (IR), Delayed Recall (DR), and the visuospatial domain from Addenbrooke’s Cognitive Examination (ACE) III. Unadjusted effect was performed using Mann Whitney U and adjustments were made using General Linear Model to examine the association between each visuo‐cognitive test and demographic factors.

**Result:**

In the total sample of 1271 participants, there were 620 females and 651 males. Mean age of females and males is 60.2±8.68 & 63.69 ±9.61 respectively. Mean years of education in females was 14.36 with a standard deviation of ±3.92. Mean years of education in males was 15.33 with a standard deviation of ±4.07. The correlation between age and MTCF Copy Time was positive, whereas with visuospatial span, visual attention, MTCF Copy, MTCF DR, and visuospatial domain of ACE III age was negatively correlated. Females performed better on tests of VA, MTCF IR, and the visuospatial domain on ACE III. There was a positive correlation between education and visuospatial span, visual attention, MTCF Copy, IR, DR, and visuospatial domain of ACE III. A negative correlation was found between education and MTCF Copy time and no significant association of the variables with MTCF IR Time.

**Conclusion:**

Age and education influenced various visuo‐cognitive components, while gender had specific impacts on visual attention and MTCF measures. These findings provide insights for early diagnosis, tailored interventions, and further investigations into cognitive health.